# Trends of the leprosy control indicators in Benin from 2006 to 2018

**DOI:** 10.1186/s12889-020-09341-w

**Published:** 2020-08-18

**Authors:** Ronald Sètondji Gnimavo, Parfait Djossou, Ghislain Emmanuel Sopoh, Gimatal Esaï Anagonou, Yves Thierry Barogui, Akpéédjé Anita Carolle Wadagni, Jean-Gabin Houezo, Roch Christian Johnson

**Affiliations:** 1 Centre de Dépistage et de Traitement de la Lèpre et de l’Ulcère de Buruli « Raoul et Madeleine Follereau, Pobè, Bénin; 2 Institut Régional de Santé Publique- Comlan Alfred Quenum, Université d’Abomey Calavi, Ouidah, Bénin; 3 Fondation Raoul Follereau, Paris, France; 4 Centre Inter Facultaire de Formation et de Recherche en Environnement pour le Développement Durable, Université d’Abomey Calavi, Abomey Calavi, Bénin; 5 Programme National de Lutte contre la Lèpre et l’Ulcère de Buruli, Ministère de la Santé, Cotonou, Bénin; 6 Centre de Dépistage et de Traitement de l’Ulcère de Buruli de Lalo, Lalo, Bénin

**Keywords:** Epidemiology, Leprosy, Benin

## Abstract

**Background:**

Leprosy, or Hansen’s disease, is a chronic infectious disease caused by *Mycobacterium leprae*. The purpose of this study was to describe the epidemiological characteristics of leprosy in Benin from 2006 to 2018.

**Methods:**

This descriptive retrospective study included data from January 2006 to December 2018. The data of all patients treated in the leprosy treatment centres (LTCs) of the Republic of Benin were obtained from the LTC registers and analysed using Stata/SE 11.0 software. Quantum GIS (Geographic Information System) version 2.18.23 software was used for mapping. The main indicators of leprosy were calculated according to the World Health Organization (WHO) recommendations.

**Results:**

During the study period, a total of 2785 (annual average of 214) new cases of leprosy were diagnosed. The median age of the patients was 38 years, with extremes ranging from 6 to 88 years. The sex ratio (males/females) was 1.18 (1509/1276). The departments of Plateau, Atacora, and Zou were the most endemic; their leprosy detection rate per 100,000 population during these thirteen years were 6.46 (479/7414297), 5.38 (534/9932880) and 5.19 (526/10134877), respectively. The leprosy detection rate declined from 3.8 to 1.32 per 100,000 inhabitants. The proportion of paediatric cases varied from 8.56 to 2.67% as the proportion of multibacillary forms increased from 72.95 to 90%. From 2006 to 2018, 622 leprosy patients detected had grade 2 disability (G2D) at screening, indicating an average rate of 5.06 (622/122877474) cases with G2D per million population. The proportion of grade 2 disabilities increased from 21.23 to 32% during the study period. The majority of new leprosy cases among foreign-born persons were Nigerian (85.71%). The completion of multidrug therapy (MDT) for paucibacillary (PB) and multibacillary (MB) leprosy cases ranged from 96.36 to 95.65% and from 90.53 to 94.12%, respectively.

**Conclusion:**

In Benin, leprosy remains a major health challenge; it is important to revitalize the epidemiological surveillance system to achieve its elimination by 2030.

## Background

Leprosy, or Hansen’s disease, is a chronic infectious disease caused by *Mycobacterium leprae* [1, 2]. It mainly affects the skin, respiratory tract epithelium, peripheral nerves and eyes. Moreover, in the absence of early treatment, it could result in visible and irreversible deformities associated with significant stigmatization and alteration of the individual’s quality of life [3, 4]. Leprosy, like other neglected tropical diseases, is one of the oldest diseases and is most prevalent among people living in poor communities [5]. With the introduction of multidrug therapy (MDT), the prevalence of leprosy has dropped sharply, and it is below the threshold of one case per 10,000 inhabitants in several countries. Therefore, the World Health Organization (WHO) has stated that the goal of eliminating leprosy as a public health problem has been achieved. Despite these major achievements, new cases of leprosy continue to be detected in all WHO regions, meaning that leprosy persists, and its transmission continues in these different regions despite the availability of effective and free antibacterial treatment [6]. In addition, it is estimated that more than 3 million people worldwide live with disabilities caused by leprosy [7]**.** Therefore, while leprosy is a widespread public health challenge, it is particularly challenging for developing countries such as Benin, whose health systems are most often confronted with insufficient human, material and financial resources. In response to the threat against human health posed by leprosy, the WHO developed a new strategy in April 2019 to accelerate the reduction of the disease burden of leprosy towards a “leprosy-free world” by 2030 [8]. To achieve effective and efficient control, break the leprosy transmission chain and ultimately attain “a Benin free of leprosy”, it is important to review the evolution of leprosy over time and space in Benin. This study, which also serves as an evaluation, will, on the one hand, allow the different actors involved in epidemiological surveillance to know the current situation of leprosy in Benin, to identify the gaps, to define new priorities; and, on the other hand, it will allow the development or readjustment of control strategies in order to implement activities to considerably reduce the leprosy-related disease burden in the population. The objectives of this study were to describe the epidemiological characteristics of leprosy in Benin over time and space from 2006 to 2018 based on key WHO indicators, such as indicators of case detection, case management and follow-up.

## Methods

### Setting

The study was conducted in Benin in eight leprosy treatment centres (LTCs) and peripheral-level health facilities managed by specialized health workers (called leprosy supervising nurses (LSNs)) who work in collaboration with the National Leprosy and Buruli Ulcer Control Programme (NLBUCP).

**Fig. 1:**
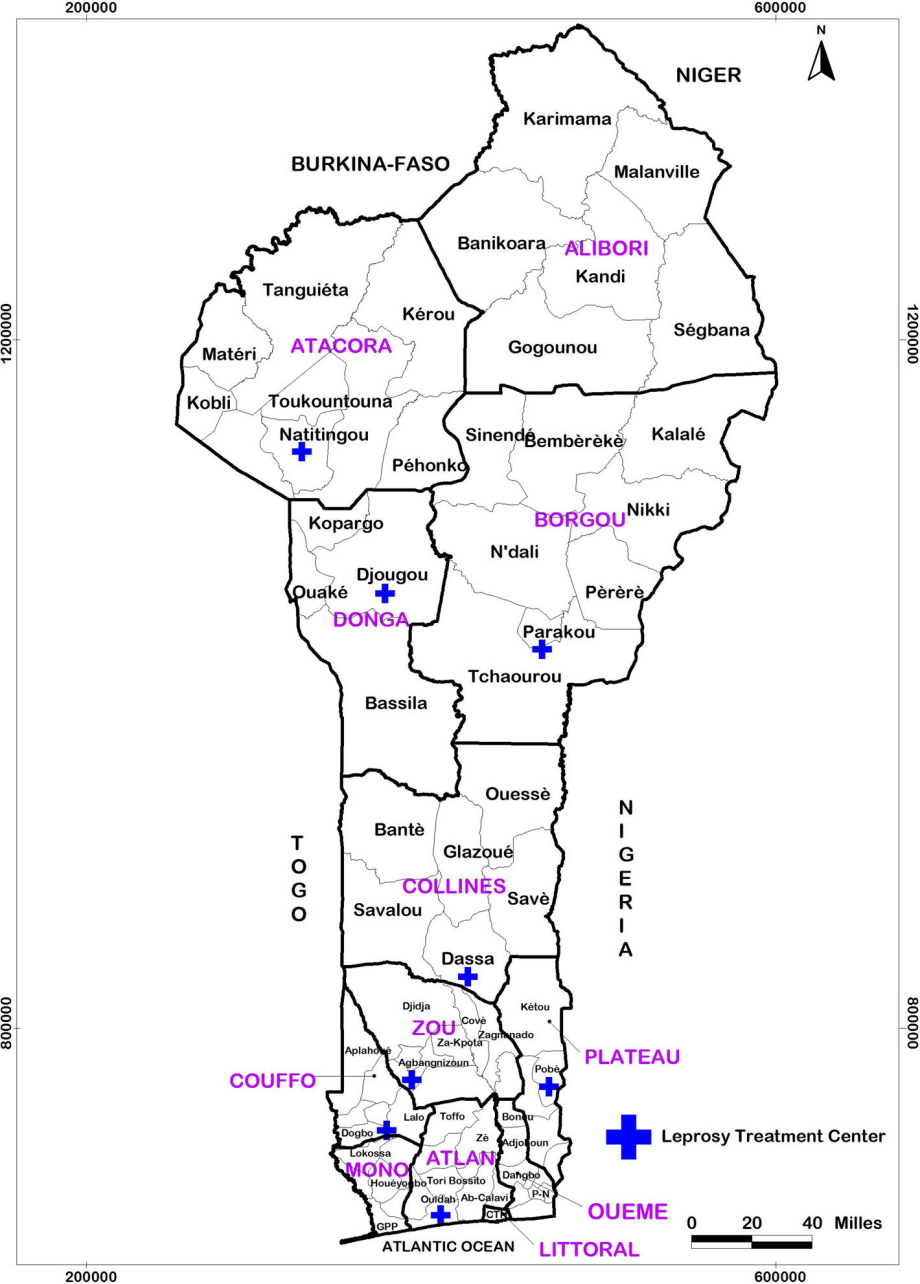
Administrative map of Bénin, showing the location of the leprosy treatment centres in the departments

LTCs are considered peripheral-level structures. They are under the technical supervision of the NLBUCP and are strategically located in Pobè, Ouidah, Madjrè, Davougon, Dassa-Zoumé, Parakou, Djougou and Natitingou in the Republic of Benin (Fig. 1); LTCs work in collaboration with peripheral-level health facilities.

The LTCs specialize in the management of people affected by leprosy; leprosy management includes the screening, diagnosis, hospitalization (when needed), medical treatment (MDT), surgical treatment (when needed), psychosocial care and community reintegration of patients.

The NLBUCP, established in 1989, works in collaboration with the health system structures at all levels. Teams of LSNs, trained by the NLBUCP for the detection and management of leprosy, are established in all 77 districts of the country and ensure the coordination between the LTCs and the other peripheral health centres.

The NLBUCP of Benin ensures the development of policies and strategies, the mobilization of resources for the implementation of activities, monitoring and evaluation and the organization of research activities. The NLBUCP of Benin organizes a formative supervision of the LTCs and LSNs on a periodic basis (every six months) to validate the leprosy cases detected and strengthen the skills of all the actors involved in the epidemiological surveillance of leprosy in Benin. The NLBUCP also ensures the monitoring and evaluation of the different activities in the fight against leprosy in Benin.

In its commitment to reducing the leprosy-related disease burden in Benin, the NLBUCP of Benin has faced several difficulties. These include the permanent decrease in the material and financial resources allocated for leprosy control every year since the elimination threshold set by WHO was reached as well as the retirement of several qualified agents (specialized in dermato-leprology) who had mastered leprosy surveillance and their replacement by new agents. This situation has caused a delay in the implementation of activities, irregularity in formative supervision and an insufficient motivation mechanism for the peripheral level staff.

### Study type and period

We conducted a descriptive retrospective study using data from the leprosy patient files kept by the LSNs and the LTCs. The period considered was from January 1st, 2006 to December 31st, 2018.

### Sampling process

We made a census of all the records of all persons affected by leprosy who were cared for by the LSNs and the various CTALs during the study period.

### Variables

The variables collected for the study included patient date of birth, date of screening or clinical diagnosis, sex, operational classification of leprosy type (paucibacillary or multibacillary), degree of disability at diagnosis according to the WHO classification (0, 1, 2), start and end dates of treatment, department of origin and patient nationality.

### Data processing and analysis

The data were entered into Epi Info software version 7.2.1.0 after checking for completeness and consistency. The qualitative data were coded. Given the lack of missing data and duplicates in the information collected, we analysed the data collected with Stata/SE 11.0 software. Proportions were calculated for the qualitative variables. Medians and extremes were determined for quantitative variables with asymmetric distributions. QGIS software version 2.18.23 was used to produce maps for the detection of new leprosy cases in each department of Benin from 2006 to 2018. A linear trend curve was used to assess the evolution of the main indicators over time.

The main leprosy indicators were determined based on recommendations made by the World Health Organization [9]. In addition, data from the 4th national population census, updated for each geographic area and accounting for the natural rate of increase, were used to calculate the detection rates [10].

According to the detection quality indicators and based on the available data, the following were determined:

- The number and rate of new cases.
- The percentage of MB (multibacillary) leprosy cases among all new cases detected.
- The percentage of female leprosy cases among all new cases detected.
- The percentage of paediatric cases (children under 15 years of age) among all new cases detected.
- The proportion of leprosy cases with grade 2 disabilities (G2D).
- The percentage of foreign-born persons with leprosy among all new cases detected.

According to the quality of case management and follow-up and taking into consideration data availability, the following indicators were determined:

- Percentage of PB (paucibacillary) and MB leprosy cases who completed their treatment within the normal duration of treatment (completion of multidrug therapy for PB and MB leprosy).

## Results

### Sociodemographic and clinical characteristics

From 1st January 2006 through 31st December 2018 (a 13-year period), a total of 2785 new cases of leprosy were recorded, corresponding to an annual average of 214 cases. There were 1509 (54%) males and 1276 (46%) females with a sex ratio of 1.18.

The median age of people affected by leprosy was 38 years, and the age range was from 6 to 88 years. Table 1 shows the distribution of new leprosy cases detected in Benin from 2006 to 2018 according to their sociodemographic characteristics.

**Table 1:** Socio-demographic and clinical characteristics of new leprosy cases detected in Benin from 2006 to 2018

Variables	Frequency (n)	Percentage (%)
**Age**		
< 15 years	283	10.16
≥ 15 years	2502	89.84
**Gender**		
Male	1509	54.18
Female	1276	45.82
**Type of leprosy**		
Paucibacillary	720	25.85
Multibacillary	2065	74.14
**Grade 2 Disability**		
Yes	622	22.33
No	2163	77.67
**Country of origin**		
Benin	2680	96.23
Foreign Cases	105	3.77

### Case detection indicators

**Fig. 2:**
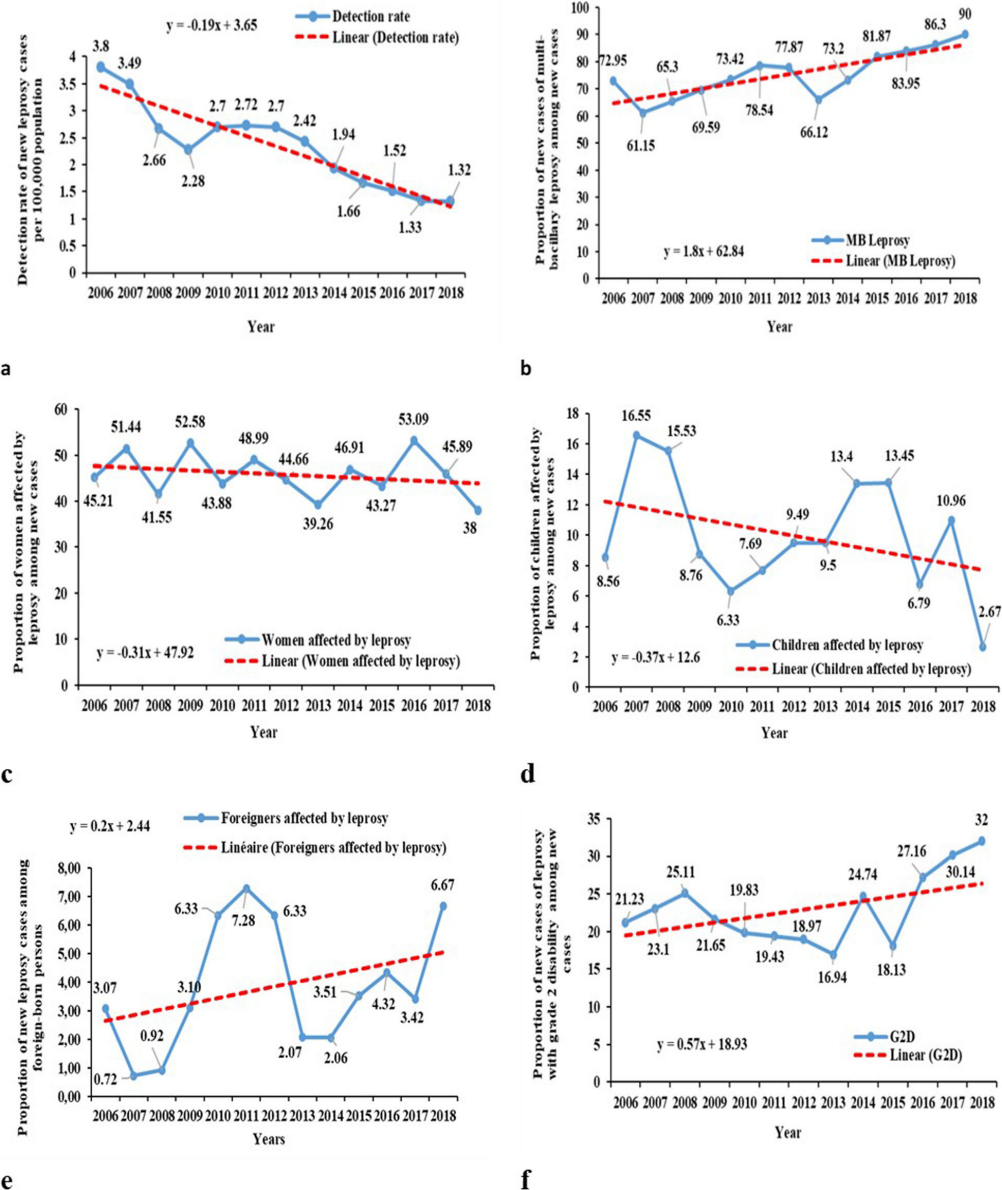
Trends of main quality of detection indicators, from 2006 to 2018, in Benin. (**a**) Detection rate of new leprosy cases per 100,000 population; (**b**) Proportion of multi-bacillary leprosy among new cases; (**c**) Proportion of women affected by leprosy among new cases; (**d**) Proportion of children affected by leprosy among new cases; (**e**) Proportion of new leprosy cases among foreign-born persons; (**f**) Proportion of grade 2 disability among new cases

The overall mean leprosy detection rate during the study period was 2.27 (2785/122,877,474) per 100,000 inhabitants. The leprosy detection rate in Benin decreased from 3.80 (292/7,680,151) to 1.32 (150/11,362,269) per 100,000 inhabitants between 2006 and 2018. (Fig. 2a).

The proportion of multibacillary forms increased from 72.95 to 90% (Fig. 2b).

The proportion of female leprosy cases decreased from 45.21 to 38% (Fig. 2c).

The proportion of paediatric leprosy cases decreased from 8.56 to 2.67% (Fig. 2d).

The proportion of leprosy cases in foreign-born persons increased from 3.07 to 6.67% (Fig. 2e).

The majority of new leprosy cases among foreign-born persons were Nigerian (85.71%). Table 2 shows the distribution of new leprosy cases by country of origin.

**Table 2:** Evolution of leprosy cases among foreign-born persons detected from 2006 to 2018 in Benin

Foreign countries	Number and (percentage) of new leprosy cases detected in Benin among foreign-born persons per year													
	2006	2007	2008	2009	2010	2011	2012	2013	2014	2015	2016	2017	2018	Total
**Nigeria**	6	2	1	5	15	14	14	5	2	6	6	4	10	90 (85.21)
**Togo**	1	0	1	0	0	1	1	0	1	0	1	1	0	7 (6.67)
**Niger**	1	0	0	0	0	2	1	0	1	0	0	0	0	5 (4.76)
**Burkina Faso**	1	0	0	1	0	0	0	0	0	0	0	0	0	2 (1.9)
**Ghana**	0	0	0	0	0	1	0	0	0	0	0	0	0	1 (0.95)
**Total**	9	2	2	6	15	18	16	5	4	6	7	5	10	105 (100)

From 2006 to 2018, 622 leprosy patients detected had G2D at screening, resulting in an average rate of 5.06 (622/122877474) cases with G2D per million population. The proportion of cases with grade 2 disabilities (G2D) ranged from 21.33 to 32% (Fig. 2f).

**Fig. 3:**
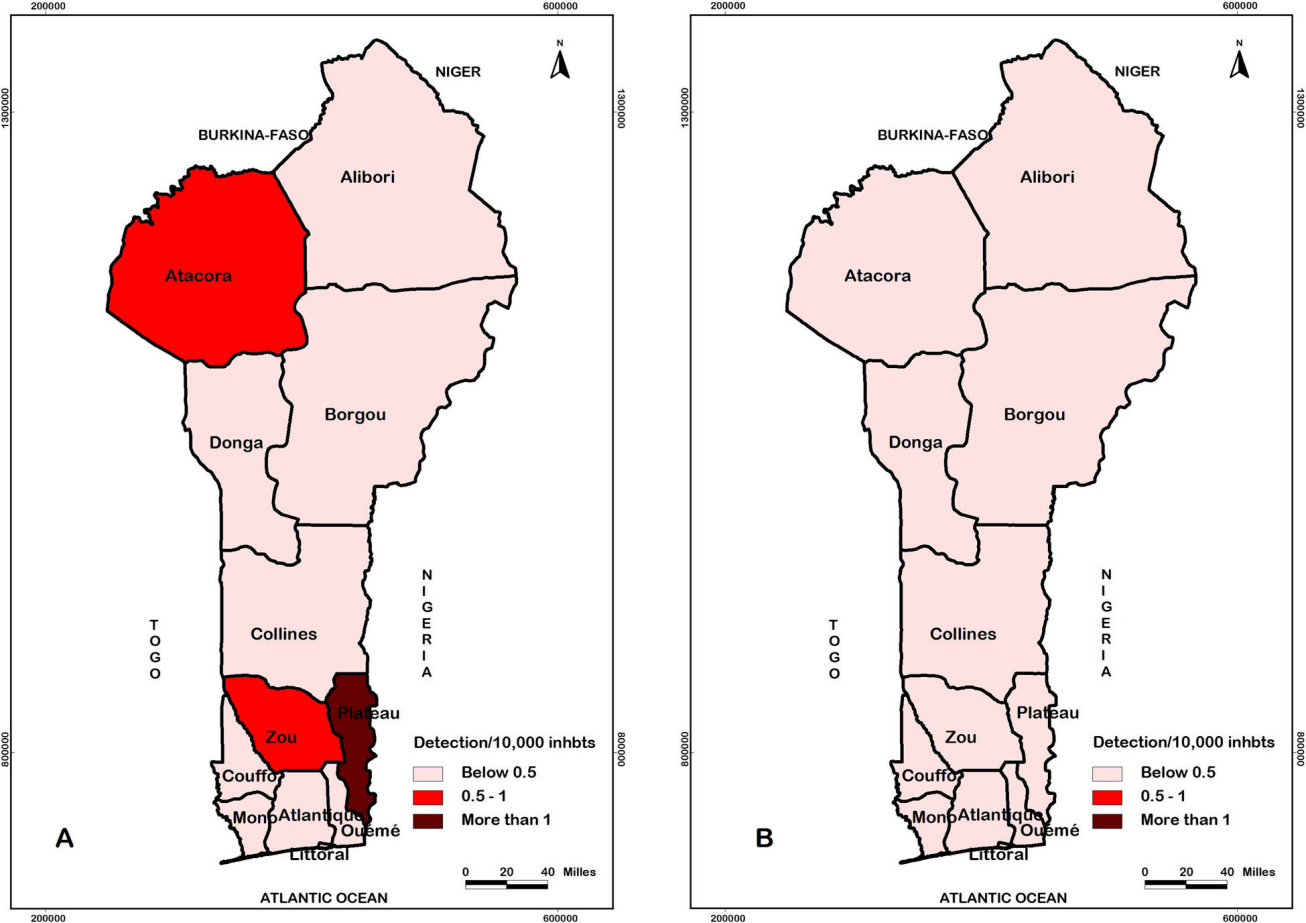
Evolution of the epidemiological situation of leprosy in Benin from 2006 (**a**) to 2018 (**b**). The detection per 10,000 inhabitants was represented on the maps by range of colours, from bright red to dark red. Lighter is the colour, least endemic the locality is; darker is the colour, more endemic the locality is

The departments of Plateau, Atacora, and Zou were the most endemic; their leprosy detection rates per 100,000 population during these thirteen years were 6.46 (479/7414297), 5.38 (534/9932880) and 5.19 (526/10134877), respectively. The general trend observed tended towards a decrease in the number of new leprosy cases detected across all departments of Benin. Table 3 and Fig. 3 show the evolution of the epidemiological situation of leprosy in Benin from 2006 (Fig. 3a) to 2018 (Fig. 3b).

**Table 3:** Distribution of new leprosy cases detected in Benin by department of origin from 2006 to 2018

Departments		Number and (detection rate) of new leprosy cases per 100,000 population											
	2006	2007	2008	2009	2010	2011	2012	2013	2014	2015	2016	2017	2018
Atlantique	11 (1.21)	12 (1.27)	7 (0.72)	6 (0.6)	19 (1.83)	10 (0.93)	14 (1.26)	6 (0.43)	12 (1.01)	5 (0.41)	3 (0.24)	6 (0.46)	8 (0.59)
Littoral	8 (1.06)	4 (0.51)	1 (0.12)	4 (0.48)	5 (0.58)	4 (0.45)	6 (0.65)	5 (0.74)	3 (0.30)	2 (0.2)	3 (0.29)	1 (0.09)	1 (0.09)
Mono	3 (0.73)	2 (0.47)	4 (0.91)	3 (0.66)	9 (1.93)	4 (0.83)	6 (1.20)	2 (0.40)	5 (0.94)	2 (0.36)	0 (0)	1 (0.17)	0 (0)
Couffo	23 (3.98)	22 (3.57)	7 (1.1)	18 (2.73)	12 (1.76)	17 (2.42)	22 (3.03)	33 (4.45)	19 (2.45)	29 (3.63)	19 (2.30)	11 (1.29)	9 (1.02)
Zou	46 (6.74)	64 (9.07)	46 (6.31)	28 (3.72)	28 (3.60)	49 (6.1)	52 (6.27)	51 (5.99)	38 (4.29)	36 (3.94)	30 (3.18)	30 (3.08)	28 (2.78)
Collines	19 (3.12)	12 (1.90)	4 (0.61)	8 (1.19)	17 (2.45)	17 (2.37)	11 (1.48)	15 (2.09)	11 (1.39)	6 (0.73)	5 (0.59)	12 (1.38)	13 (1.44)
Ouémé	7 (0.84)	8 (0.93)	3 (0.34)	6 (0.65)	8 (0.84)	10 (1.02)	5 (0.49)	7 (0.64)	4 (0.37)	1 (0.09)	10 (0.87)	1 (0.08)	5 (0.41)
Plateau	59 (12.74)	70 (14.62)	60 (12.13)	27 (5.28)	49 (9.28)	37 (6.78)	35 (6.21)	35 (5.61)	21 (3.50)	20 (3.22)	25 (3.90)	18 (2.72)	23 (3.37)
Borgou	27 (3.28)	12 (1.41)	17 (1.93)	5 (0.55)	11 (1.17)	17 (1.75)	15 (1.50)	22 (1.83)	23 (2.15)	8 (0.73)	4 (0.35)	10 (0.85)	6 (0.49)
Alibori	10 (1.69)	1 (0.16)	9 (1.42)	3 (0.46)	2 (0.3)	1 (0.14)	2 (0.28)	2 (0.23)	3 (0.39)	2 (0.25)	5 (0.61)	1 (0.12)	0 (0)
Atacora	55 (8.80)	59 (9.13)	39 (5.84)	47 (6.82)	42 (5.89)	50 (6.79)	42 (5.53)	33 (4.29)	38 (4.69)	36 (4.30)	34 (3.93)	32 (3.58)	27 (2.93)
Donga	15 (3.77)	10 (2.43)	20 (4.70)	33 (7.51)	20 (4.41)	13 (2.77)	27 (5.57)	26 (4.79)	13 (2.52)	18 (3.37)	17 (3.08)	18 (3.16)	20 (3.40)
Total	283 (3.68)	276 (3.47)	217 (2.64)	188 (2.21)	222 (2.53)	229 (2.52)	237 (2.53)	237 (2.37)	190 (1.90)	165 (1.6)	155 (1.45)	141 (1.33)	140 (1.23)

### Case management and follow-up indicators

**Fig. 4:**
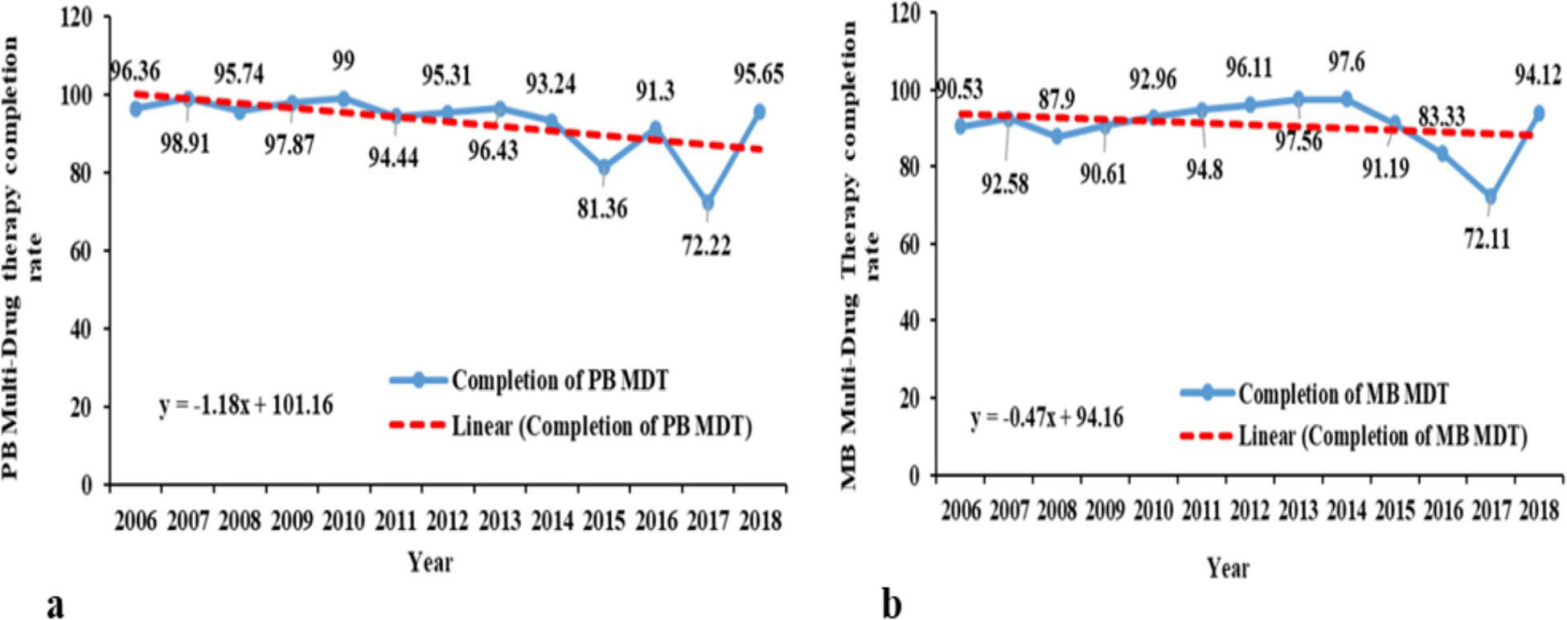
Trends of main quality of management and follow-up indicators from 2006 to 2018, in Benin. (**a**) PB Multi-Drug Therapy completion rate; (**b**) MB Multi-Drug Therapy completion rate

The completion of multidrug therapy (MDT) for paucibacillary (PB) leprosy cases was above 91% during the study period, except for the years 2015 and 2017. These years were marked by a substantial drop to 81 and 72%, respectively (Fig. 4a).

The percentage of MB leprosy cases that completed treatment within the normal treatment timeframe increased from 90.53 to 94.12% (Fig. 4b).

## Discussion

### Key results

This retrospective study of leprosy records made it possible to describe the evolution of leprosy control in Benin according to the key WHO indicators. The detection rate and the proportion of paediatric cases declined from 3.8 to 1.32 per 100,000 inhabitants and from 8.56 to 2.67%, respectively; however, there was an increase in the proportions of multibacillary forms of grade 2 disabilities from 72.95 to 90% and 21.23 to 32%, respectively. Furthermore, a magnitude of inequality is noted from one department to another, with Plateau (6.46 cases per 100,000 inhabitants), Atacora (5.38 cases per 100,000 inhabitants), and Zou (5.19 cases per 100,000 inhabitants) being the most endemic. The majority of new leprosy cases among foreign-born persons were Nigerian (85.71%). The multidrug therapy (MDT) completion rates for paucibacillary (PB) and multibacillary (MB) leprosy cases ranged from 96.36 to 95.65% and from 90.53 to 94.12%, respectively.

Due to its exhaustive nature, this analysis provides an idea of the epidemiological characteristics of leprosy in Benin. Benin has achieved elimination of leprosy as a public health problem according to the WHO definition, and case detection indicators continue to decrease slowly. Nevertheless, many indicators suggest ongoing transmission and a need for further action.

### Limitations

One of the limitations of this study and one of the weaknesses of the NLBUCP surveillance system are the lack of data on contact cases, retreatment, resistance to treatment and leprosy reactions for all cases. Indeed, these indicators provide information on the quality of detection, treatment and follow-up of cases. The availability of this information would have allowed us to make a holistic analysis of all the key indicators as defined by WHO in addition to this overview of the epidemiological situation of leprosy in Benin. It would therefore be important for the NLBUCP to organize the surveillance system to routinely capture these data, even if they seem to not be directly useful for control activities.

### Sociodemographic characteristics

The average age of our patients was close to that reported by Keïta et al. in Mali and by Ouedraogo et al. in Burkina Faso [11, 12]. This implies that leprosy mainly affects young people at economically active phases of their lives, and this is of great concern because the absence of early disease diagnosis could result in disabilities that can exert negative repercussions on the individual, his or her family and the economy of the country in the long term [13, 14]. Consequently, leprosy keeps its victims in the vicious circle of poverty. According to the United Nations Development Programme (UNDP) World Report published in 2019, Benin was counted as a developing country. It is characterized by a low human development index (a composite index that measures the average level achieved in three fundamental dimensions of human development: long and healthy life, knowledge and decent standard of living). Indeed, in Benin, the human development index was 0.52, with an estimated multidimensional poverty rate (percentage of the population with a deprivation score of at least 33%) of 66.8% [15]. According to Schmitt et al., the probability of being poor was almost five times higher in leprosy patients than in unscathed individuals [16]. 

### Case detection indicators

The equation for the linear trend curve indicated that the leprosy detection rate decreased, on average, by 0.19 units per 100,000 inhabitants each year. This observed decreasing trend in the detection of new leprosy cases is consistent with that observed by the WHO worldwide (average annual decrease of 2% between 2007 and 2016) [8]. In Burkina Faso, Ouédraogo et al. also reported a decrease in new leprosy cases from 913 to 187 between 2000 and 2015 [17]. Similarly, Simionato de Assis et al. reported a decrease in the detection rate of new leprosy cases (4.3% per year) over a 13-year time series from 2003 to 2015 in a border region of Latin America [5], and Crouzat et al. reported a decrease in the number of new leprosy cases, from 7.6 to 3.96 per 100,000 inhabitants, between 1991 and 2011 in Noumea (New Caledonia) [18]. Although the decrease in the detection rate of new cases of leprosy observed in this study corroborates the data in the literature, we believe that it does not reflect the real epidemiological situation of leprosy in Benin. From our point of view, this decrease could result from an underreporting of cases, thus reflecting inadequacies of the entire health system for community screening, as well as the loss of experienced health workers with expertise in precise identification or diagnosis of leprosy cases. It could also be a direct consequence of the low commitment of decision-makers and partners who favour so-called “priority” diseases and diseases with epidemic potential, resulting in a progressive loss of support and funding for leprosy control activities. This might also suggest that policy makers, donors and governments wrongly equate elimination (as a public health problem) with eradication. Thus, they may have erroneously assumed that their efforts to rid the world of leprosy have achieved their objective [19, 20].

Based on the operational classification of leprosy according to the WHO, the multibacillary form, which is the most contagious, was the main form observed in our study. According to the equation for the linear trend curve, this form has undergone an average annual increase of 1.8% between 2006 and 2018. This increase in the proportion of multibacillary leprosy cases reflects the concept that late diagnosis leads to very high levels transmission of leprosy at the community level. A high proportion of MB leprosy cases has also been reported by several authors [21–23]. This may be because high-prevalence regions have mainly PB cases, while in low-prevalence regions, MB forms are more frequently identified [24]. This seems to justify the involvement of immunological factors. As the number of potentially contagious patients in hyperendemic areas is greater, the probability of an individual receiving a sufficiently high bacterial load to cause an infection before adulthood is greater in those regions. Consequently, PB forms will often be more commonly diagnosed in these areas. However, in low endemicity areas, individuals are less often exposed to the bacillus, so infection occurs later, and thus, the proportion of MB cases is higher [25, 26].

Official reports around the world continue to show a disparity between the number of male and female patients diagnosed with leprosy. In our study, both sexes were affected, but there was a predominance of male cases. This predominance was similar to that generally observed in several previous studies [23, 27]. This male predominance could be explained by the low status of women and the economic dependence of women on men [28]. This is even more plausible since, in Sub-Saharan Africa, economic power is most often held by men, which would also give them the opportunity to attend health facilities more frequently than women [29]. However, our results are contrary to the female predominance reported by other authors in the literature [12, 14].

New cases of leprosy in children under 15 years of age are linked to the bacillus transmission chain in the community and the existence of an active transmission site [9, 22]. In our study, the proportion of paediatric cases decreased by an average of 0.37% each year. Despite this downward trend, the proportion of cases reported by our study was above the WHO’s desired threshold of 0 cases. This situation indicates that until 2018, there was a persistence of infection transmission within the communities.

For the proportion of cases with G2D, an average annual increase of 0.57% was observed. Furthermore, the proportions reported by our study from 2006 to 2018 were well above the 5% threshold generally accepted by the WHO. The same is true for the average grade 2 disability rate, which is above the WHO key target for 2030 (less than one new case with G2D per million inhabitants). The number of new leprosy cases with G2D is an indicator that reflects the early detection of cases. Indirectly, it also provides information on other factors that influence case detection, such as the level of community awareness of leprosy, the ability of health staff to recognize early signs and symptoms and, to some extent, the quality of leprosy control services [9].

The increase in the proportion of G2D indicates that an increasing number of leprosy cases in Benin were detected late. Unfortunately, late diagnosis leads to continuous transmission and an increased risk of disability [30]. Our findings are consistent with those reported by others [14, 17]. This epidemiological situation is an indication that Benin is far from achieving the goal of the WHO Global Leprosy Strategy 2016–2020, which targets zero children affected by leprosy with visible deformities. Therefore, it would be important for stakeholders to conduct studies to understand the individual- and health system-related factors that could explain the high prevalence of G2D despite the existence of leprosy control services. These results are in contrast with those published by the WHO, which reported an overall significant decrease in cases with G2D in 2017 [8]. The discordance could be related to the early case detection campaigns implemented by some national programmes.

Findings from this study showed an average annual increase of 0.20% in new leprosy cases among foreign-born individuals. A high proportion of leprosy cases among foreign-born persons may result from easy access to leprosy services for foreigners but might also reflect the quality of the services provided to them as a vulnerable group [9]. The majority of the foreign-born patients originated from Nigeria, one of Benin’s neighbouring countries. This could be explained partly by the fact that Nigeria is one of the 23 WHO priority countries due to its very high leprosy-related disease burden (more than 1000 new cases of leprosy reported annually for more than 10 years). Alternatively, these results could be explained by the fact that, in the border region, the population flows between the two countries are high but the services for the control of cross-border communicable diseases are almost non-existent.

It would therefore be important for the NLBUCP of Benin to work with the actors in charge of leprosy control in these five countries (Benin, Nigeria, Togo, Niger and Burkina Faso) towards the establishment of a system of surveillance and cross-border management. This cooperation will make it possible to develop cross-border collaboration to harmonize screening and monitoring strategies and then to coordinate the referral and counter-referral of cross-border patients.

The Plateau, Atacora and Zou departments were the most endemic in Benin. This epidemiological situation could be an indication of the existence of a disparity between the different departments of Benin, which needs further research to be clarified. However, several hypotheses can be discussed:

- This pattern could be due to an intense circulation of *M Leprae* in these localities, or more likely, this situation could reflect the operational factors related to leprosy screening in these departments. It could also reflect the high susceptibility of individuals living in these communities to leprosy due to their low socio-economic conditions, which reflects the intensity of poverty in these departments. In both cases, the identification of these different zones offers avenues of research for the actors involved in the epidemiological surveillance system. It would therefore be important for them to initiate studies to identify all the factors associated with the high prevalence of leprosy in the Plateau, Atacora and Zou departments.

In the current context of insufficient human, material and financial resources allocated to the control of leprosy, the identification of these three departments should help for better planning and allocation resource and implementation of adequate leprosy control strategies. In other words, these different departments must become a priority target for the actions of the NLBUCP of Benin.

### Case management and follow-up indicators

The management of leprosy does not stop at the time of diagnosis or after the administration of MDT because the disease can still progress, causing reactivations; therefore patients with disability will need lifelong care. With regard to case follow-up, our results showed an average annual decrease of 1.18 and 0.47%, respectively, in MDT completion rates for PB and MB leprosy cases, but most of the recorded completion rates were well above the 85% threshold generally accepted by the WHO [9]. The decrease in completion rates for PB and MB leprosy cases found in our study is contrary to that reported by Salah et al., who found completion rates close to 100% in their study in Oman in 2017 [31]. This reflects the need for those involved in epidemiological surveillance to identify the likely causes of treatment non-completion among leprosy patients and to implement strategies to encourage patients to complete their treatment in order to avoid complications related to treatment discontinuation.

A meta-analysis of the evolutionary trend of the main leprosy indicators in Benin allows us to conclude that there is a low endemic leprosy situation that is causing delays in diagnosis. It would therefore be important for Benin’s NLBUCP to develop innovative strategies to achieve the key targets of the WHO global strategy for 2030. A participative approach is recommended to solve the different priority problems identified during the data analysis, and we encourage the epidemiological surveillance actors who are at the decision-making level to elaborate a 5-year strategic plan. A package of five key strategies is essential to achieve the WHO’s key targets. These include:

- Advocacy to draw the attention of the politico-administrative authorities and technical and financial partners to the need to increase the budget allocated to the PNLLUB.
- Training and capacity building of health personnel to increase their ability to detect the early signs and symptoms of the disease.
- Promotion of active case and contact screening in high-risk communities.
- Creation of a system of surveillance and cross-border management with the actors in charge of the fight against leprosy in the countries bordering Benin.
- Targeted awareness, education and communication initiatives for behaviour change.
- To ensure the implementation, progress and status of the activities resulting from this strategic plan and the achievement of the stated objectives, we propose that the NLBUCP develop a coordination, monitoring and periodic evaluation mechanism.

## Conclusion

Benin, like several countries in the world, has achieved the leprosy elimination target of less than one case per 10,000 inhabitants, but the disease remains a major health challenge due to the young age of the population affected by the disease, the high proportion of multibacillary leprosy cases, the persistence of transmission of the infection and, above all, the increase in number of people with grade 2 disabilities among the newly detected cases. In view of this, it is imperative to strengthen the epidemiological surveillance of the disease through information, education and communication sessions; additionally, the adoption of new strategies that encourage the early and accurate detection of new leprosy cases in at-risk populations is also essential.

## Data Availability

The datasets used and/or analysed in the current study are available from the corresponding author upon request with valid justification.

## Abbreviations

CNERS: Comité National d’Ethique pour la Recherche en Santé
LTC: Leprosy treatment centres
G2D: Grade 2 disability
LSN: Leprosy supervising nurses
MDT: Multidrug therapy
MB: Multibacillary
NLBUCP: National Leprosy and Buruli Ulcer Control Programme
PB: Paucibacillary
QGIS: Quantum Geographic Information System
WHO: World Health Organization
